# Role of Extracellular Vesicles of Stem Cells from Human Exfoliated Deciduous Teeth in Osteogenesis

**DOI:** 10.3390/ijms26125841

**Published:** 2025-06-18

**Authors:** Rio Shibata, Ryo Kunimatsu, Shota Ito, Tomohiro Ogasawara, Shintaro Ogashira, Ayaka Nakatani, Kodai Rikitake, Ayaka Odo, Akira Hirabae, Io Koyanagi, Takaharu Abe, Tomoka Hiraki, Shuzo Sakata, Yuki Yoshimi, Kotaro Tanimoto

**Affiliations:** 1 Department of Orthodontics and Craniofacial Developmental Biology, Graduate School of Biomedical and Health Sciences, Hiroshima University, Hiroshima 734-8553, Japan; ksty@hiroshima-u.ac.jp (R.S.); anakatan@hiroshima-u.ac.jp (A.N.); d224959@hiroshima-u.ac.jp (A.H.); koyanagi@hiroshima-u.ac.jp (I.K.); takabe@hiroshima-u.ac.jp (T.A.); tomoka1012@hiroshima-u.ac.jp (T.H.); shuzosakata@hiroshima-u.ac.jp (S.S.); tkotaro@hiroshima-u.ac.jp (K.T.); 2 Department of Orthodontics, Division of Oral Health and Development, Hiroshima University Hospital, Hiroshima 734-8553, Japan; shota0313@hiroshima-u.ac.jp (S.I.); t-ogasawara@hiroshima-u.ac.jp (T.O.); milk595@hiroshima-u.ac.jp (S.O.); shuna-s0102@hiroshima-u.ac.jp (K.R.); ayakaodo@hiroshima-u.ac.jp (A.O.); yukimihsoy@hiroshima-u.ac.jp (Y.Y.)

**Keywords:** EVs, culture supernatant, SHED, MSCs, bone regeneration

## Abstract

The tissue regenerative potential of the liquid component of mesenchymal stem cells has gained significant attention. Stem cells from human exfoliated deciduous teeth-conditioned medium (SHED-CM), which is often extracted during orthodontic treatment, promotes bone regeneration. However, further investigation is warranted to determine which component of SHED-CM affects bone regeneration. Therefore, we focused on the extracellular vesicles contained in SHED-CM (SHED-EVs) and aimed to study their effects on osteoblasts. SHED-EVs were isolated using a pellet-down EV extraction kit and identified using transmission electron microscopy and NanoSight. SHED-EVs were added to human calvarial osteoblasts (HCOs), and cell proliferation and migration ability were examined with Incucyte^®^ and BrdU. Alkaline phosphatase (ALP) expression was confirmed using real-time PCR and ALP quantification. The bone differentiation potential was examined using Alizarin Red S (ARS) staining. SHED-EVs promoted proliferation and migration of HCOs. Real-time PCR and ALP quantification results demonstrated that HCOs cultured with SHED-EVs exhibited increased ALP expression. ARS staining revealed that SHED-EVs promoted bone differentiation of HCOs. These results suggest that SHED-EVs promote cell proliferation and migration and bone regeneration of osteoblasts, highlighting their potential in the development of bone regeneration therapies.

## 1. Introduction

Mesenchymal stem cells (MSCs) were first identified in the bone marrow as colony-forming cells capable of differentiating into osteoblasts, adipocytes, and chondrocytes [1]. MSCs, which are present in various tissues and maintain homeostasis during remodeling and tissue repair, are used not only to restore hematopoietic function and treat autoimmune diseases but also to repair various tissue injuries, including those of the skin, bone, cartilage, heart, and nerve [2,3,4,5,6]. Most clinical trials use bone MSCs (BMSCs); however, this is problematic because it requires bone marrow puncture, which is burdensome for the patient [7,8,9]. Technologies for the isolation and culture of MSCs from dental pulp, periodontal ligament, and alveolar bone have been developed in the field of dentistry, thereby contributing to research and development for practical regenerative medicine of dental pulp and periodontal tissue [10]. The presence of dental pulp stem cells (DPSCs) and stem cells from human exfoliated deciduous teeth (SHEDs) in the dental pulp has been reported, and these stem cells have been implicated in the regeneration of the dentin-pulp complex [11,12]. These stem cells can be manipulated in vitro by colony formation methods; moreover, hard tissue formation can be induced by transplantation using tissue engineering techniques. Tissue engineering is a concept in which regeneration is achieved through the appropriate fusion of three elements: stem cells that regenerate tissue, scaffolds that guide stem cells about the size and shape of the tissue or organ to be regenerated, and signaling molecules that promote stem cell growth and differentiation [13,14].

We previously reported the effects of BMSCs, DPSCs, and SHEDs on bone regeneration. Using parietal bone-deficient immunodeficient mice, DPSCs and SHEDs were shown to have bone regenerative potential similar to that of BMSCs [15]. In vitro, SHEDs demonstrated higher cell proliferative capacity than did BMSCs and DPSCs and higher expression of basic fibroblast growth factor (bFGF) and bone morphogenetic protein (BMP)-2 genes following the induction of bone differentiation [16]. This indicated that SHEDs may be useful in bone regeneration therapy. In addition, the paracrine action of MSCs has recently attracted attention, suggesting the possibility of inducing tissue regeneration via cytokine therapy using stem cell culture supernatants (MSC-conditioned medium (CM)) [17]. Therefore, we studied the effects of SHED-CM on bone regeneration. SHED-CM grafts reportedly exhibit bone regenerative effects [18]. However, further clarification is warranted regarding the effects of liquid components of SHED-CM on cell proliferation and bone regeneration.

Accordingly, here, we focused on the extracellular vesicles (SHED-EVs) contained in SHED-CM. EVs are small lipid bilayer membrane structures with a diameter of 30–150 nm, secreted by cells and found in various body fluids, including blood, urine, saliva, and breast milk. EVs contain proteins, mRNA, miRNA, DNA, and lipids that are taken up by other cells and presumably play important roles as intercellular messengers [19]. MSC-EVs are thought to possess the biological functions of stem cells, and their therapeutic effects have been reported [20,21,22,23,24,25,26,27]. Additionally, the bone regenerative potential of BMSC-EVs and DPSC-EVs has been reported [28,29,30]. However, there have been no reports of bone regeneration using SHED-EVs. As SHEDs are easy to isolate in a minimally invasive manner and are juvenile cells, investigating the effects of SHED-EVs is important. Additionally, since the origin and uptake mechanisms of exosomes remain unclear, optimizing high-purity EV extraction is essential. Therefore, this study aimed to investigate the effects of SHED-EVs on cell proliferation and bone regeneration and examine the mechanisms and extraction methods of SHED-EVs.

## 2. Results

### 2.1. Identification of EVs Extracted from SHED

**Figure 1:**
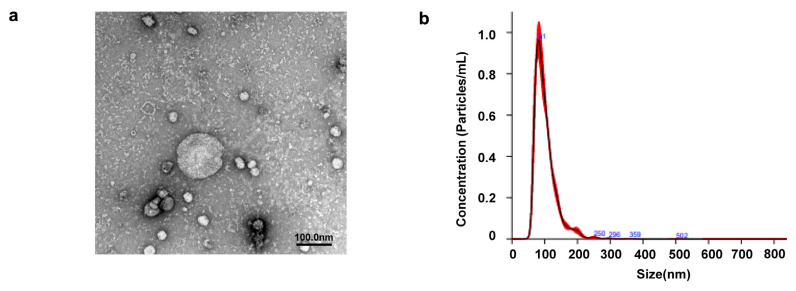
(**a**) Transmission electron microscopy image of extracellular vesicles of stem cells from human exfoliated deciduous teeth (SHED-EVs). (**b**) Nanoparticle tracking analysis (NanoSight) of SHED-EVs.

TEM of the SHED-EVs revealed a spherical morphology of 100 nm (Figure 1a). Nanoparticle tracking analysis (NTA) confirmed that the average particle size of the SHED-EVs was 102.2 nm (Figure 1b).

The detailed SHED-EV NanoSight results are shown in Table 1. The median of SHED-EVs was 79.9 ± 3.2 nm and the standard deviation (SD) 36 ± 1.1 nm, D10 (where 10% of the total was entered) 70.6 ± 1.4 nm, D50 (where 50% of the total was entered) 93.5 ± 1.4 nm, and D90 (where 90% of the total was entered) 142.3 ± 2.1 nm. Finally, the particle size (concentration) of SHED-EVs was 5.22 × 10^8^ ± 1.52 × 10^7^ (particle/mL).

### 2.2. Cell Proliferation and Migration Ability of SHED-EVs

**Figure 2:**
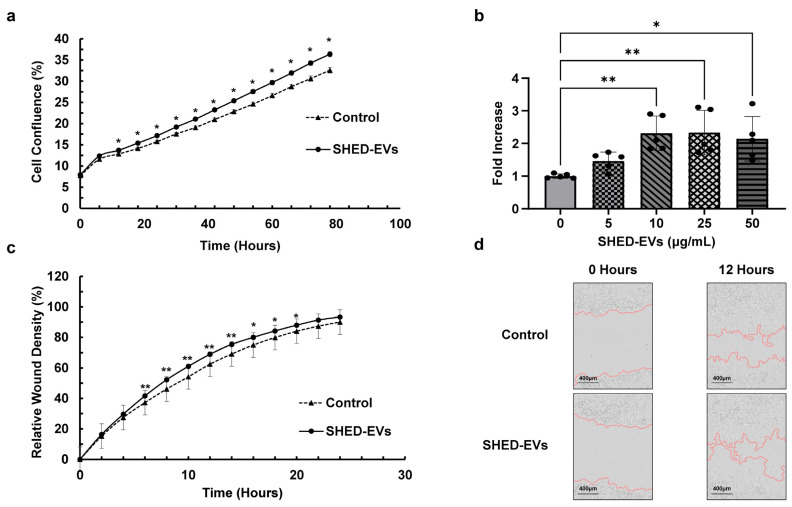
(**a**) Observation of cell proliferative potential through a live cell imaging system (Incucyte^®^) (*n* = 40 per group, * *p* < 0.05). (**b**) Observation of cell proliferative potential with the BrdU Cell Proliferation Assay (*n* = 5 per group, * *p* < 0.05, ** *p* < 0.01). (**c**) Scratch wound assay with Incucyte^®^ (*n* = 24 per group, * *p* < 0.05, ** *p* < 0.01). (**d**) Scratch wound assay. HCO images obtained 0 and 12 h after scratching with Incucyte^®^. HCO, human calvarial osteoblast.

HCOs were cultured, SHED-EVs were added 24 h later, and cell density was observed using Incucyte^®^. A significant difference was observed after 12 h in the SHED-EV group compared with the control group (Figure 2a). In the BrdU Cell Proliferation Assay, the absorbance was also significantly higher in the SHED-EV group at concentrations of 10, 25, and 50 μg/mL than in the control group. No significant differences were found for the group at a concentration of 5 μg/mL and among groups added at concentrations of 10, 25, and 50 μg/mL (Figure 2b). The HCOs were incubated, confirmed to be 100% confluent after 16 h, and then scratched. SHED-EVs were added at a concentration of 10 μg/mL when replacing the medium containing 1% fetal bovine serum (FBS) and observed every 2 h for 24 h using Incucyte^®^. There was a significant increase in cell migration in the SHED-EV group over that in the control group 6–20 h later (Figure 2c,d).

### 2.3. Bone Regeneration Potential of SHED-Evs

**Figure 3:**
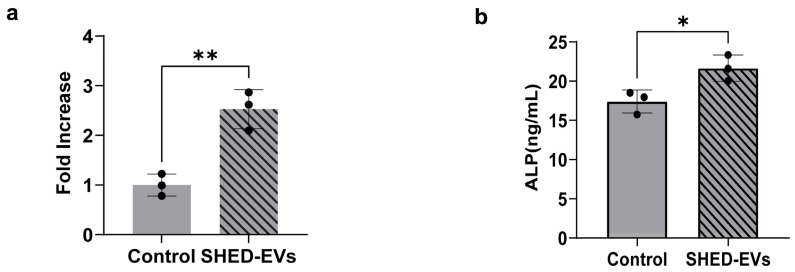
(**a**) Real-time polymerase chain reaction was performed to confirm alkaline phosphatase (ALP) expression (*n* = 3 per group, ** *p* < 0.01). (**b**) ALP quantification. The absorbance at 450 nm was measured. (*n* = 3 per group, * *p* < 0.05).

HCOs were cultured with SHED-EVs. RNA was collected 24 h later, and real-time polymerase chain reaction (RT-PCR) was performed to confirm ALP expression, which was significantly higher in the SHED-EV group than in the control group (Figure 3a). HCOs were cultured with SHED-EVs, and the culture supernatant was collected 48 h later for ALP quantification using enzyme linked immunosorbent assay (ELISA). The protein expression levels of ALP were significantly higher in the SHED-EV group than in the control group (Figure 3b).

**Figure 4:**
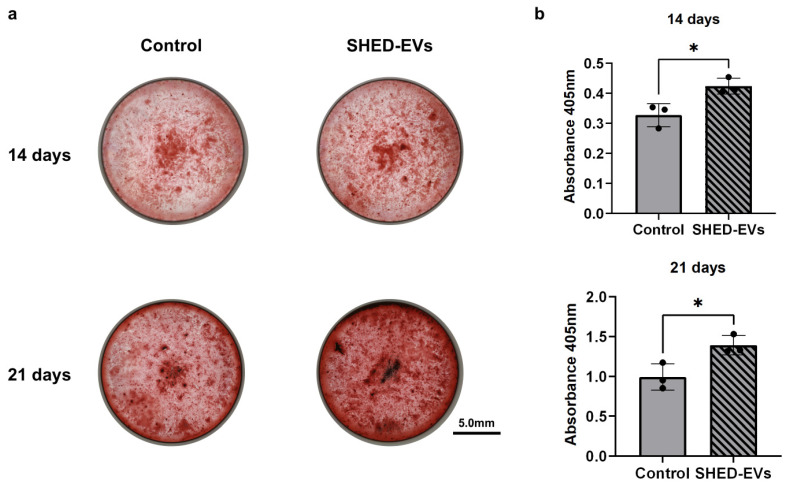
(**a**) Alizarin red S staining. Human calvarial osteoblasts (HCOs) were cultured in bone differentiation medium with SHED-EVs for 14 and 21 days and stained with Alizarin red S. (**b**) Alizarin red S staining. Dissolved after Alizarin red S staining, and the absorbance was measured at 405 nm (*n* = 3 per group, * *p* < 0.05). SHED, stem cells from human exfoliated deciduous teeth; EV, extracellular vesicle.

Furthermore, HCOs were cultured in bone differentiation medium with SHED-EVs for 14 and 21 days and stained with Alizarin red S, which was more enhanced in the SHED-EV group than in the control group both at 14 and 21 days (Figure 4a). When dissolved and the absorbance was measured, the SHED-EV concentrations of 10 μg/mL were significantly higher in the SHED-EV group than in the control group, as well as in the groups incubated for 14 and 21 days (Figure 4b).

## 3. Discussion

Regenerative medicine is a treatment method that attempts to regenerate lost functions by utilizing embryonic, induced pluripotent, and somatic stem cells in dysfunctional organisms. In particular, MSCs have been studied extensively because they can differentiate not only into adipose and bone tissues, which are derived from the mesoderm, but also into visceral and nervous tissues, which are derived from the endoderm and ectoderm. The paracrine action of MSCs has recently attracted considerable attention, and cell-free therapy using factors such as culture supernatants and EVs in the culture supernatants has also been studied [31]. Cell-free therapy offers improved safety due to the low risk of immune rejection and tumor formation following transplantation, and reduced cost and time owing to the elimination of cell culture. Platelet-rich plasma (PRP) therapy promotes healing of damaged tendons, ligaments, and muscles by injecting plasma enriched with platelet-derived EVs [32]. Stem cell-derived EVs are reportedly more effective than steroids in reducing inflammation in coronavirus disease-2019 and chronic infections [33]. Possible methods for utilizing exosomes include the creation of EVs overexpressing therapeutic molecules, such as mRNA, miRNA, and proteins to increase the therapeutic efficacy, and development of DDS technology to specifically transport them to target tissues [34,35]. The SHEDs used in this study may need to be extracted during orthodontic treatment, and their collection is useful because it is convenient for patients in clinical settings.

In this study, isolated SHED-EVs were confirmed using TEM and NTA to have particle sizes in the 100–150 nm range. This is similar to the identification of DPSC-EVs [30]. We were able to extract EVs from SHED-CM. In this study, exosomes were extracted from SHED-CM using the pellet-down method and the Total Exosome Isolation Kit. Progressive research on EVs has led to the utilization of various methods of exosome extraction [36,37,38]. Ultracentrifugal extraction is the most used method; however, alternative methods are warranted for facilities without ultracentrifuges. Ultrafiltration and size-exclusion chromatography extraction are recommended for better accuracy. The extraction solution used in this study can extract EVs by adding half of the concentrated culture supernatant, incubating overnight, and then centrifuging at 10,000× *g* for 1 h. It is advantageous in being a simple method that does not require special machinery such as an ultracentrifuge. However, it has the disadvantage of containing more EVs and proteins other than EVs than other isolation methods. The EVs extracted with size exclusion chromatography were photographed using TEM, and the small structures around them were reduced, suggesting that they were proteins or aggregates. As the possibility of these proteins acting on cell proliferation and bone regeneration cannot be ruled out, more precise extraction methods are warranted in future studies.

In this study, observations with a live cell imaging system demonstrated that the SHED-EV group had significantly increased cell proliferative capacity than the control group (* *p* < 0.05). When BrdU was performed, a significant increase in the cell proliferative capacity was observed in the SHED-EV group (* *p* < 0.05, ** *p* < 0.01). The results of the scratch assay using the live cell imaging system demonstrated a significant enhancement of the cell migration capacity in the SHED-EV group (* *p* < 0.05, ** *p* < 0.01). Our findings indicate that SHED-EVs significantly enhance the proliferative and migratory capacities of HCOs. These results are consistent with those on BMSC-EV and DPSC-EVs additions [29,30], as well as other reports on the proliferative and migratory potential of osteoblasts with the addition of MSC-EVs [39,40].

ALP promotes bone formation by degrading pyrophosphate, a calcification inhibitor [41]. Hypophosphatasia is a calcification disorder caused by the accumulation of pyrophosphate, which does not break down owing to reduced ALP activity. In the oral cavity, premature loss of deciduous teeth is presumably attributed to weakening of the periodontal ligament bond due to cementum malformation. ALP is produced in the early stages of osteoblast development and is commonly found on cell surfaces, bones, and matrix vesicles of calcified cartilage [42]. Being present in various tissues of almost all organisms, it is widely used as a biomarker and an important marker of osteoblast differentiation. In this study, we noted a significant increase in the ALP expression and concentration in the SHED-EV group compared to that in the control group using quantitative PCR and ELISA (* *p* < 0.05, ** *p* < 0.01). In addition, the SHED-EV group was stained more intensely in both the 14- and 21-day cultures. These results suggest that SHED-EVs promote the early stages of osteoblast differentiation and bone formation (* *p* < 0.05, ** *p* < 0.01). The findings are consistent with those of BMSCs and DPSCs [29,30]. Considering that BMSCs require a bone marrow puncture, which places a significant burden on the patient, SHEDs can be considered useful as they are easily accessible and their procurement is non-invasive.

MicroRNAs (miRNAs) in EVs have attracted increasing attention. In 2007, MC/9 cell-derived EVs were reported to contain approximately 1300 mRNAs and 121 miRNAs [43]. In 2010, miRNA function in EVs was noted in the receiving cells [44,45,46]. Bone regenerative effects of exosomes overexpressing specific miRNAs have also been confirmed [47]. Most circulating miRNAs reside in exosomes [48]. EVs can provide patient information, prognostic information regarding diseases, and real-time pathological information [49,50,51]. EVs can also be used for a minimally invasive diagnostic test known as liquid biopsy, or for treating diseases through EV implantation [52]. Therefore, EV-associated miRNAs are not only considered biomarkers for diagnosis and prognosis but also potential carriers of therapeutic agents [43,53]. Future analyses of the miRNAs contained in SHED-EVs will be useful for elucidating detailed bone regeneration mechanisms and applying them to bone regeneration therapy.

This study had some limitations. First, although the methods used to isolate SHED-EVs have been established, it was difficult to independently investigate exosomes and miRNA present in SHED-EVs. Therefore, to elucidate the effects of SHED-EVs on bone regeneration, miRNA-sequence analysis must be performed to delineate the aspects of exosomes and miRNA contained in SHED-EVs.

Further studies to identify factors such as miRNA that promote bone regeneration are also essential. Although osteoblasts were used in this study, the tissue regeneration efficacy and safety of SHED-EVs in vivo were not evaluated. Therefore, further studies using in vivo methods are needed.

## 4. Materials and Methods

### 4.1. Isolation and Culture of SHED and HCOs

Dental pulp tissue was collected from deciduous teeth extracted for orthodontic treatment. Collection, isolation, and culture were performed according to the method described by Gronthos et al. [11]. This study was conducted in strict compliance with the regulations of epidemiological research at the Hiroshima University Hospital (E-20-2).

The extracted deciduous teeth were transported in phosphate-buffered saline (PBS; PHC Holdings Corporation, Tokyo, Japan) containing 100 mM amphotericin (MP Biomedicals, Santaana, CA, USA), ethanol (Hayashi Pure Chemical, Osaka, Japan), and isodine (Kenei Seiyaku, Osaka, Japan). The periodontal ligament of the extracted tooth was detached as much as possible with a scalpel, and the tooth was divided using osteoclastic forceps (Natsume Corporation, Tokyo, Japan). The amputated deciduous dental pulp was treated with 4 mg/mL collagenase (Thermo Fisher Scientific, Waltham, MA, USA), 3 mg/mL dispase (Thermo Fisher Scientific, Waltham, MA, USA) containing Minimum Essential Medium Eagle (α-MEM) (Sigma-Aldrich, St. Louis, MO, USA), and incubated at 37 °C until the pulp was degraded. The supernatant was then centrifuged at 1800 rpm for 5 min, aspirated, and suspended in 20% FBS (Central Link, Tokyo, Japan), 0.024% kanamycin (Meiji Seika Pharma, Tokyo, Japan), 0.05% penicillin (Meiji Seika Pharma, Tokyo, Japan), and 0.1% amphotericin-containing α-MEM; seeded in 35-mm dishes, and incubated at 37 °C under 5% CO_2_ conditions. When the cells reached confluence, they were detached and passaged in PBS containing 0.25% trypsin (Nacalai Tesque Corporation, Kyoto, Japan) and 1 mM ethylene diamine tetraaceticacid (EDTA) (FUJIFILM Wako Pure Chemical Corporation, Osaka, Japan). After the first passage (P1), the cells were cultured in α-MEM containing 10% FBS and the above antibiotics under 37 °C and 5% CO_2_ conditions. Cells P2–P9 were used in this study.

Primary HCOs were obtained from the ScienCell Research Laboratories. Before the start of culture, 100-mm dishes were coated (2 μg/cm^2^) with poly-L-lysine stock solution (ScienCell Research Laboratories, San Diego, CA, USA) and allowed to settle overnight. The poly-L-lysine-coated dish was washed twice with sterile water and filled with 10 mL of complete medium (Osteoblast Medium [ObM], ScienCell Research Laboratories, San Diego, CA, USA). The frozen cells were thawed in a water bath at 37 °C and transferred to medium. The cells were cultured under 37 °C and 5% CO_2_ conditions. The medium was changed the next day, once every 3 days until 70% confluence until passage, and daily thereafter. PolyL-lysine-coated dishes were prepared 1 day before passaging. Complete medium, 0.05% trypsin/EDTA (ScienCell Research Laboratories, San Diego, CA, USA), Trypsin Neutralization Solution (ScienCell Research Laboratories), and Dulbecco’s phosphate-buffered saline (ScienCell Research Laboratories) were brought to room temperature, and the cells were peeled off and passaged. Cells were seeded into the culture vessels at a density of 5000 cells/cm^2^. After the first passage (P1), the cells were cultured in ObM containing 5% FBS (ScienCell Research Laboratories, San Diego, CA, USA) under 37 °C and 5% CO_2_ conditions. Cells P2–P7 were used in this study.

### 4.2. Isolation and Purification of EVs Derived from SHED

SHEDs were cultured, and the medium was replaced with serum-free α-MEM when they reached 80% confluency; the culture supernatant was collected 48 h later. The collected supernatant was centrifuged at 300× *g* for 5 min, after which it was further centrifuged at 2000× *g* for 30 min. The supernatant was collected, concentrated 40-fold on an Amicon Ultra-15 centrifugal filter (Merck Millipore, Darmstadt, Germany), and collected using the pellet-down method. In detail, half the volume of Total Exosome Isolation Reagent (Thermo Fisher Scientific, Waltham, MA, USA) was added, incubated overnight at 4 °C, and centrifuged at 10,000× *g* for 1 h at 4 °C. The supernatant was aspirated to avoid sucking the EVs clumps attached to the bottom of the tube, suspended in PBS, and stored at −80 °C until use. Exosomes suspended in PBS were measured using a micro bicinchoninic acid assay (BCA) protein assay kit (Thermo Fisher Scientific, Waltham, MA, USA) to evaluate the EV concentration.

### 4.3. Identification of EVs Derived from SHED

#### 4.3.1. Transmission Electron Microscopy

The morphology of the EVs was observed using transmission electron microscope (TEM). SHED-EVs were suspended in PBS and dropped onto a carbon-film grid for 10 s. Excess water was wiped off, and negative staining was performed using 2% uranyl acetate. Images were obtained at 100 kV using a JEM-1400Flash (JEOL, Tokyo, Japan).

#### 4.3.2. Nanoparticle Tracking Analysis of EVs

The particle distribution and particle number concentration of EVs were measured using NTA. The EVs were diluted in PBS and measured using NanoSight NS300 (Malvern Panalytical, Almelo, The Netherlands). The results were analyzed using NanoSight NTA3.3 software (Malvern Panalytical, Almelo, The Netherlands).

### 4.4. Cell Proliferation Test

#### 4.4.1. Observation of Cell Proliferative Potential Using Live Cell Imaging System

HCOs were seeded in 24-well plates at a density of 5000 cells/cm^2^. After 24 h, viability was confirmed, the medium was changed and SHED-EVs at a concentration of 10 μg/mL were added simultaneously; the cell density of HCOs was observed every 6 h for 72 h on Incucyte^®^ (Sartorius AG, Göttingen, Germany). The results were analyzed using an Incucyte S3 system (Sartorius AG).

#### 4.4.2. Evaluation of the Cell Proliferative Potential via BrdU

HCOs were seeded in 96-well plates at a density of 5000 cells/cm^2^ and incubated at 37 °C under 5% CO_2_ conditions. After reaching 80% confluence, the FBS concentration in the medium was changed stepwise to 0%, and at 0%, SHED-EVs were added at concentrations of 0, 5,10, 25, and 50 μg/mL, cultured for 24 h, and then subjected to BrdU Cell Proliferation Assay (Merck KGaA, Darmstadt, Germany) was performed. Cell counts were evaluated by measuring the absorbance at 370 nm according to the manufacturer’s instructions.

#### 4.4.3. Scratch Wound Assay

HCOs were seeded into 96-well plates at a density of 62,500 cells/cm^2^ and incubated at 37 °C for 16 h under 5% CO_2_ conditions; 100% confluence was confirmed and scratched using the Incucyte^®^ 96-Well Woundmaker Tool Kit (4475; Sartorius AG). FBS dropped to 1% at medium change, SHED-EVs were added at a concentration of 10 μg/mL and observed for 24 h on the Incucyte^®^ for analysis. Results were analyzed with the Incucyte^®^ S3 system.

### 4.5. Evaluation of Alkaline Phosphatase Expression

#### 4.5.1. Real-Time Polymerase Chain Reaction

HCOs were seeded in 6-well plates at a density of 5000 cells/cm^2^ and incubated at 37 °C under 5% CO_2_ conditions. When 80% confluence was reached, the FBS concentration in the medium was reduced stepwise, and at 0%, SHED-EVs were added at a concentration of 10 μg/mL, incubated for 24 h, and RNA was collected. RT-PCR was performed using the Thermal Cycler Dice^®^ Real Time System (Takara Bio, Shiga, Tokyo) and SYBR Green Master Mix (TOYOBO, Osaka, Japan) at 95 °C for 1 min, 40 cycles at 95 °C for 15 s and 60 °C for 31 min. Glyceraldehyde-3-phosphate dehydrogenase (*GAPDH*) was used as the housekeeping gene for mRNA. *ALP* gene expression was analyzed, and the relative expression levels were calculated using the 2^−ΔΔCt^ method (Table 2).

#### 4.5.2. ALP Quantification

HCOs were seeded in 6-well plates at a density of 10,000 cells/cm^2^, FBS was reduced stepwise at 80% confluence, SHED-EVs were added at a concentration of 10 μg/mL at 0%, and supernatants were collected after 48 h of incubation. A human ALP ELISA kit (Abcam, Cambridge, UK) was used to measure the absorbance at 450 nm according to the manufacturer’s instructions to assess the ALP concentration.

#### 4.5.3. Alizarin Red S Staining

HCOs were seeded in 24-well plates at a density of 10,000 cells/cm^2^, and the medium was changed every 2 days. When 80% confluence was reached, the medium was replaced with bone differentiation medium comprising DMEM supplemented with 10 mM β-glycerophosphate, 0.2 mM ascorbic acid, 100 mM dexamethasone, and 10% FBS, and SHED-EVs were added at concentrations of 10 μg/mL and 25 μg/mL. The same concentration of SHED-EVs was added simultaneously, and the medium was changed every three days. The cells were cultured for 14 and 21 days, fixed in 95% neutral buffered formalin solution for 10 min, and stained at room temperature with alizarin red S (ARS) solution from a calcification evaluation set (PG Research, Tokyo, Japan). Calcification was evaluated by dissolving the calcified nodule lysate and measuring the absorbance at 405 nm.

### 4.6. Statistical Analysis

Results are presented as mean ± standard deviation (SD); *t*-test was used for comparisons between the two groups and Tukey’s test for multiple comparisons. The significance level was set at *p* < 0.05 or *p* < 0.01.

## 5. Conclusions

In conclusion, in this study, we isolated EVs from SHED-CM. The addition of isolated EVs to HCO increased cell proliferation and migration. Moreover, alkaline phosphatase expression in HCOs supplemented with SHED-EVs increased, and the osteodifferentiation capacity of HCOs was enhanced. Our findings suggest that SHED-EVs increase the cell proliferative, migration, and bone regeneration capacities, thus highlighting their potential in the development of bone regeneration therapies.

## Figures and Tables

**Table 1:** Detailed results of the nanoparticle tracking analysis of SHED-EVs.

Parameters	Merged Data (nm)	Standard Error of the Mean (nm)
Mean	102.2	1.4
Mode	79.9	3.2
SD	36	1.1
D10	70.6	1.4
D50	93.5	1.4
D90	142.3	2.1

**Table 2:** Primer sequence.

Gene	Sequence (5′→3′)
GAPDH (Forward)	GGCCTCCAAGGAGTAAGACC
GAPDH (Reverse)	AGGGGTCTACATGGCAACTG
ALP (Forward)	AGAATCTGGTGCAGGAATGG
ALP (Reverse)	CATGAGATGGGTCACAGACG

## Data Availability

The data presented in this study are available on request from the corresponding author.

## Abbreviations

The following abbreviations are used in this manuscript:

MSC	mesenchymal stem cells
DPSC	dental pulp stem cells
SHEDs	stem cells from human exfoliated deciduous teeth
BMSCs	bone mesenchymal stem cells
BMP	bone morphogenetic protein
ALP	alkaline phosphatase
ARS	Alizarin Red S
